# Heart rate variability as a biomarker in patients with Chronic Chagas Cardiomyopathy with or without concomitant digestive involvement and its relationship with the Rassi score

**DOI:** 10.1186/s12938-022-01014-6

**Published:** 2022-06-28

**Authors:** Luiz Eduardo Virgilio Silva, Henrique Turin Moreira, Marina Madureira de Oliveira, Lorena Sayore Suzumura Cintra, Helio Cesar Salgado, Rubens Fazan, Renato Tinós, Anis Rassi, André Schmidt, J. Antônio Marin-Neto

**Affiliations:** 1 Division of Cardiology, Department of Internal Medicine, Ribeirão Preto Medical School, University of São Paulo, Av. Bandeirantes, 3900, Ribeirão Preto, São Paulo 14048-900 Brazil; 2 Department of Physiology, Ribeirão Preto Medical School, University of São Paulo, Ribeirão Preto, Brazil; 3 Department of Computing and Mathematics, Ribeirão Preto School of Philosophy, Science and Literature, University of São Paulo, Ribeirão Preto, Brazil; 4 Anis Rassi Hospital, Goiânia, Brazil

**Keywords:** Autonomic nervous system, Chagas disease, Heart rate variability, Machine learning, Rassi score

## Abstract

**Background:**

Dysautonomia plays an ancillary role in the pathogenesis of Chronic Chagas Cardiomyopathy (CCC), but is the key factor causing digestive organic involvement. We investigated the ability of heart rate variability (HRV) for death risk stratification in CCC and compared alterations of HRV in patients with isolated CCC and in those with the mixed form (CCC + digestive involvement). Thirty-one patients with CCC were classified into three risk groups (low, intermediate and high) according to their Rassi score. A single-lead ECG was recorded for a period of 10–20 min, RR series were generated and 31 HRV indices were calculated. The HRV was compared among the three risk groups and regarding the associated digestive involvement. Four machine learning models were created to predict the risk class of patients.

**Results:**

Phase entropy is decreased and the percentage of inflection points is increased in patients from the high-, compared to the low-risk group. Fourteen patients had the mixed form, showing decreased triangular interpolation of the RR histogram and absolute power at the low-frequency band. The best predictive risk model was obtained by the support vector machine algorithm (overall F1-score of 0.61).

**Conclusions:**

The mixed form of Chagas' disease showed a decrease in the slow HRV components. The worst prognosis in CCC is associated with increased heart rate fragmentation. The combination of HRV indices enhanced the accuracy of risk stratification. In patients with the mixed form of Chagas disease, a higher degree of sympathetic autonomic denervation may be associated with parasympathetic impairment.

## Introduction

More than a century after the publication of the first reports by the Brazilian physician Carlos Chagas, the pathogenesis of Chagas disease (CD) is still not fully understood by researchers and clinicians [1–3]. The infection with the protozoan *Trypanosoma cruzi* entails the acute phase of CD, which lasts about 4–8 weeks and is often asymptomatic. Then, the long-lasting—several decades—chronic phase of CD follows, and several individual outcomes occur in the development of the disease, which is considered the result of a complex host–parasite interaction involving several immune mechanisms [4].

As long as individuals with chronic CD do not exhibit cardiac or digestive clinical manifestations, they are said to harbor the indeterminate form of the disease. This form of CD accounts for asymptomatic individuals infected by the *T. cruzi* but with normal findings on the conventional 12-lead electrocardiogram (ECG) and on the radiological examinations of heart, esophagus and colon [5]. In contrast, individuals who become symptomatic or show objective signs of organic involvement are said to harbor the determinate form of the disease, usually with cardiac, digestive or mixed organic involvement [6, 7].

Individuals with Chronic Chagas Cardiomyopathy (CCC) have the most frequent and more ominous form of CD, representing the potential evolution of 40% of *T. cruzi* infected subjects. CCC entails the development of serious cardiac complications, for instance, ventricular aneurysms, systemic and pulmonary thromboembolism, heart failure and complex arrhythmias, the latter representing one of the most important risk factors for sudden death [8, 9]. Various mechanisms have been considered to be involved in the pathogenesis of CCC, including microvascular disturbances, parasite-dependent and immune-mediated myocardial damage, and cardiac dysautonomia [2, 6, 7]. Cardiac dysautonomia was demonstrated on the basis of anatomical studies showing decreased cardiac intramural neurons, and through functional approaches detecting impairment of autonomic responses to several tests, including changes in heart rate variability (HRV) [2, 10, 11]. Taking into account that HRV indices are broadly sensitive biomarkers of cardiovascular pathological conditions [12–14], it is plausible to assume that HRV bears a significant potential for risk stratification of patients with CD. Although numerous studies have reported HRV alterations in patients with CD, most of them performed only classical linear analyses and did not fully evaluate the potential of combining different HRV indices for the prognostication of patients with CD.

Considering the evidence of cardiac dysautonomia in CD and the prognostic value of HRV from other cardiovascular and systemic diseases, we hypothesized that HRV is associated with the risk of death of patients with CCC. In a previous study, we showed significant association of HRV with several morpho-functional parameters obtained from the echocardiogram carried out in patients with CD [15]. Here, we extended this analysis to seek correlations of such findings with the Rassi score, the best prognostic predictor for overall mortality in patients with CCC. Briefly, we looked at: (1) the ability of thirty linear and nonlinear HRV indices to distinguish the three classes of Rassi’s risk score, as well as the concomitance of digestive involvement associated with CCC (mixed form of chronic CD); and (2) the power of combining those HRV indices, using machine learning techniques, to predict the Rassi’s risk class of each patient.

## Results

Table 1 shows the demographic and clinical variables of the whole sample population included in the study. Data are shown for all patients as well as grouped by the Rassi score risk group.

**Table 1:** Demographic and clinical variables of the patients eligible for the study. Values are reported as median [1st, 3rd] quartiles or number of patients (percentage of patients)

	Group			
	All (*N* = 31)	Low-risk (*N* = 13)	Intermediate-risk (*N* = 10)	High-risk (*N* = 8)
Age (years)	57 [49, 68]	63 [48, 70]	54 [50, 62]	60 [49, 73]
Male gender	12 (39)	6 (46)	4 (40)	2 (25)
Body mass index (Kg/m^2^)	27.4 [23.6, 30.7]	27.7 [25.8, 30.8]	27.0 [23.3, 29.0]	26.7 [23.2, 31.2]
SBP (mmHg)	120 [108, 130]	120 [114, 129]	117 [98, 130]	120 [100, 127]
DBP (mmHg)	80 [66, 80]	80 [67, 85]	70 [66, 80]	80 [66, 80]
Digestive involvement				
Yes	14 (45)	5 (38)	2 (20)	7 (88)
No	15 (48)	7 (54)	7 (70)	1 (12)
Unknown	2 (7)	1 (8)	1 (10)	0 (0)
NYHA class				
Class I	13 (42)	10 (77)	2 (20)	1 (12)
Class II	8 (26)	3 (23)	4 (40)	1 (12)
Class III	9 (29)	0 (0)	4 (40)	5 (63)
Class IV	1 (3)	0 (0)	0 (0)	1 (12)
Medications in use				
Betablockers	24 (77)	9 (69)	9 (90)	6 (75)
Amiodarone	14 (45)	1 (8)	7 (70)	6 (75)
Amlodipine	2 (6)	1 (8)	1 (10)	0 (0)
Diuretics	25 (81)	8 (62)	10 (100)	7 (87)
Hydralazine	3 (10)	0 (0)	2 (20)	1 (12)
Nitrates	2 (6)	0 (0)	1 (10)	1 (12)
ACE-I	19 (61)	6 (46)	7 (70)	6 (75)
Angiotensin II antagonists	10 (32)	5 (38)	3 (30)	2 (25)
Rassi score variables				
NYHA III or IV	10 (32)	0 (0)	4 (40)	6 (75)
Cardiomegaly	12 (39)	0	4 (40)	8 (100)
Wall-motion abnormalities	27 (87)	9 (69)	10 (100)	8 (100)
Nonsustained VT	5 (16)	0 (0)	3 (30)	2 (25)
Low QRS voltage	8 (26)	1 (8)	3 (30)	4 (50)
Male gender	12 (39)	6 (46)	4 (40)	2 (25)

*N*: sample size; SBP: systolic blood pressure; DBP: diastolic blood pressure; NYHA: New York Heart Association; ACE-I: angiotensin-converting enzyme inhibitor; VT: ventricular tachycardia. Two patients (6%) were not in use of any of those medications. On average, patients were in use of 3.2 of those medications

Table 2 shows the median HRV indices grouped by cardiomyopathy only and mixed (cardiodigestive) forms. TINN is significantly decreased in patients with the cardiodigestive form compared to the patients with only cardiac involvement (cardiomyopathy form). There is also a strong tendency for decreased LF abs in patients with the cardiodigestive form (*p* = 0.06). No other HRV index was found to be significantly different between the two forms of CD.

**Table 2:** Median [1st, 3rd] quartiles of heart rate variability (HRV) indices in the groups of patients with the cardiomyopathy or the mixed (cardiodigestive) form of Chagas disease

	Cardiomyopathy (*N* = 15)	Cardiodigestive (*N* = 14)	*p* value
Acc Capacity (ms)	− 4.63 [− 7.92, − 3.17]	− 3.57 [− 5.07, − 2.89]	0.181
Dec Capacity (ms)	4.49 [3.02, 7.93]	3.81 [3.03, 5.34]	0.324
Porta (%)	49.9 [46.5, 51.3]	50.1 [48.6, 52.8]	0.109
Guzik (%)	49.9 [45.3, 51.5]	51.3 [48.2, 56.0]	0.117
Ehlers	− 0.34 [− 0.86, 0.57]	0.67 [− 0.38, 2.54]	0.274
DFA a1	1.10 [0.92, 1.27]	0.91 [0.71, 1.07]	0.181
AttEn	1.49 [1.20, 1.79]	1.63 [1.42, 1.83]	0.718
DispEn	4.42 [4.14, 4.67]	4.44 [4.02, 4.66]	0.840
DistEn	0.72 [0.67, 0.76]	0.66 [0.62, 0.70]	0.331
FuzzyEn	1.54 [1.37, 1.76]	1.63 [1.32, 1.73]	0.968
PermEn	2.51 [2.47, 2.55]	2.55 [2.51, 2.56]	0.081
PhaseEn	0.90 [0.87, 0.91]	0.90 [0.87, 0.91]	0.857
SampEn	1.85 [1.52, 2.02]	1.82 [1.53, 2.05]	0.868
PIP (%)	62.8 [53.7, 65.8]	63.7 [57.9, 69.2]	0.411
W0 (%)	2.7 [0.5, 3.8]	1.2 [0.8, 2.6]	0.439
W1 (%)	19.1 [12.7, 34.2]	23.1 [13.4, 32.1]	0.788
W2 (%)	61.6 [44.4, 72.2]	55.5 [48.5, 60.8]	0.424
W3 (%)	12.0 [7.4, 15.1]	16.9 [11.6, 23.8]	0.227
0 V (%)	22.0 [7.5, 32.4]	19.0 [9.9, 31.8]	0.926
1 V (%)	45.1 [34.3, 49.4]	45.0 [31.8, 50.4]	0.993
2LV (%)	4.0 [2.2, 8.3]	5.8 [2.1, 7.1]	0.994
2UV (%)	21.6 [15.5, 34.3]	24.1 [18.1, 34.3]	0.855
LF abs (ms^2^)	86.9 [44.1, 171.7]	28.5 [17.1, 97.5]	0.060
HF abs (ms^2^)	87.7 [36.4, 243.7]	51.0 [33.2, 105.8]	0.181
LF/HF	1.24 [0.59, 1.95]	0.6 [0.3, 1.8]	0.215
Mean RR (ms)	1016 [842, 1084]	977 [798, 1062]	0.522
SDNN (ms)	27.1 [19.4, 35.3]	19.8 [14.4, 24.9]	0.298
RMSSD (ms)	18.3 [11.2, 31.3]	17.2 [12.1, 20.9]	0.326
Triang Index	8.16 [5.47, 9.97]	5.68 [4.31, 7.31]	0.146
TINN (ms)	121.1 [80.1, 146.5]	78.1 [62.5, 101.6]	0.046

For the description of HRV indices, please see the text

Table 3 shows the median HRV indices grouped by the Rassi risk class. Phase entropy is decreased and PIP is increased in patients assigned to the high-risk group, when compared to patients in the low-risk group. Moreover, there is a strong tendency for increased W3 in the high-risk group (also compared to the low-risk group, *p* = 0.062). Increased PIP and W3 indicates that patients in the high-risk group have a more fragmented heart rate. No other HRV index was found to be significantly different among the three groups.

**Table 3:** Median [1st, 3rd] quartiles of HRV indices in the three Rassi risk groups

	Low	Intermediate	High	ANOVA *p* value
Acc Capacity (ms)	− 5.09 [− 7.74, − 2.94]	− 3.42 [− 4.30, − 2.79]	− 3.64 [− 4.75, − 2.70]	0.547
Dec Capacity (ms)	4.69 [3.47, 8.38]	3.14 [2.78, 4.43]	3.65 [2.93, 5.39]	0.371
Porta (%)	51.5 [49.0, 53.1]	49.3 [47.6, 50.1]	50.0 [47.6, 51.4]	0.298
Guzik (%)	51.4 [48.5, 54.9]	49.6 [47.0, 50.8]	50.8 [48.7, 55.1]	0.249
Ehlers	0.42 [-0.75, 2.65]	-0.41 [-0.85, 0.05]	0.46 [-0.13, 1.89]	0.141
DFA a1	0.98 [0.91, 1.17]	1.11 [0.85, 1.30]	0.94 [0.73, 1.12]	0.525
AttEn	1.66 [1.52, 1.94]	1.60 [1.25, 1.87]	1.38 [1.20, 1.75]	0.334
DispEn	4.46 [4.10, 4.67]	4.43 [4.20, 4.56]	4.20 [3.93, 4.68]	0.942
DistEn	0.67 [0.64, 0.76]	0.67 [0.60, 0.72]	0.69 [0.63, 0.77]	0.526
FuzzyEn	1.64 [1.33, 1.78]	1.59 [1.39, 1.71]	1.45 [1.29, 1.77]	0.992
PermEn	2.51 [2.48, 2.55]	2.51 [2.48, 2.56]	2.55 [2.53, 2.56]	0.268
PhaseEn	0.91 [0.90, 0.91]	0.88 [0.87, 0.91]	0.87 [0.86, 0.89]*	0.039
SampEn	1.87 [1.49, 2.04]	1.81 [1.51, 2.10]	1.69 [1.53, 1.92]	0.956
PIP (%)	58.2 [53.4, 63.3]	63.4 [55.8, 66.8]	66.4 [63.5, 71.2]*	0.032
W0 (%)	2.6 [1.3, 3.5]	1.1 [0.4, 3.7]	1.0 [0.6, 2.8]	0.410
W1 (%)	30.2 [18.4, 40.5]	21.5 [15.4, 31.8]	13.2 [9.7, 26.4]	0.066
W2 (%)	58.5 [44.0, 65.7]	58.3 [46.5, 63.7]	56.1 [47.7, 68.7]	0.727
W3 (%)	10.5 [8.3, 13.4]	16.5 [11.5, 22.9]	22.2 [13.3, 27.5]	0.062
0 V (%)	24.5 [10.7, 43.5]	20.7 [7.6, 31.8]	23.2 [6.2, 30.9]	0.806
1 V (%)	46.8 [39.4, 50.4]	44.9 [31.6, 48.9]	39.6 [30.3, 50.0]	0.324
2LV (%)	5.6 [2.3, 8.8]	4.5 [1.7, 8.0]	4.8 [1.3, 6.2]	0.795
2UV (%)	19.0 [12.6, 24.7]	22.3 [17.7, 34.4]	31.2 [21.3, 38.4]	0.417
LF abs (ms^2^)	57.5 [18.0, 119.7]	48.6 [19.6, 154.5]	60.5 [17.6, 104.1]	0.869
HF abs (ms^2^)	66.5 [27.2, 235.6]	47.3 [27.2, 107.1]	72.0 [38.5, 126.6]	0.789
LF/HF	1.42 [0.61, 1.82]	1.01 [0.57, 2.63]	0.53 [0.32, 1.79]	0.380
Mean RR (ms)	847 [779, 980]	1037 [843, 1111]	1059 [932, 1090]	0.132
SDNN (ms)	20.7 [16.6, 35.0]	20.1 [12.4, 27.2]	23.5 [16.1, 37.8]	0.534
RMSSD (ms)	16.7 [8.0, 26.9]	13.5 [9.2, 20.2]	20.7 [16.5, 25.1]	0.663
Triang Index	6.28 [4.73, 9.27]	5.92 [3.73, 7.97]	6.76 [4.85, 9.42]	0.776
TINN (ms)	85.9 [70.3, 132.8]	89.8 [54.7, 119.1]	89.8 [68.4, 117.2]	0.857

For the description of HRV indices, please see the text. *p < 0.05 compared to the “Low” group using Tukey’s post-hoc test

**Fig. 1:**
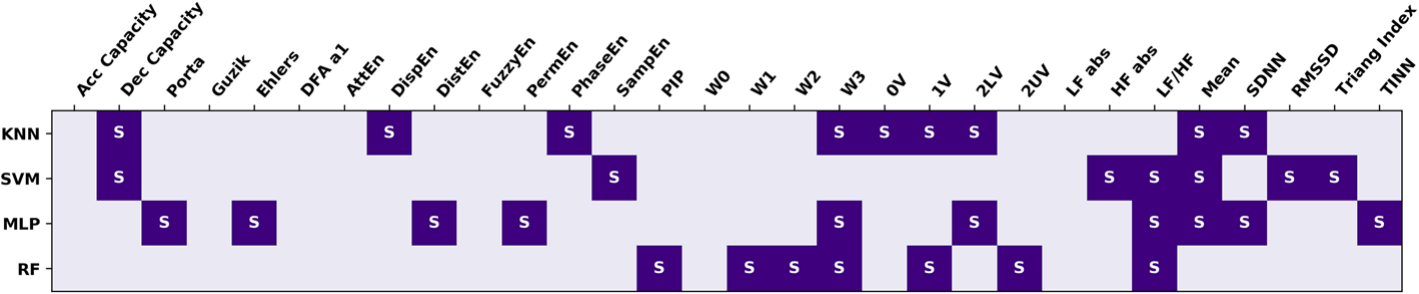
Feature selection using the four machine learning algorithms. S: selected feature; KNN: *k*-nearest neighbors; SVM: support vector machine; MLP: multilayer perceptron; RF: random forest. For the description of HRV indices (columns), please see the text

The combination of HRV indices to predict the Rassi risk group of each patient was assessed using machine learning algorithms. Figure 1 shows the results of the feature selection step for the four machine learning algorithms utilized in this work. No feature was selected by all four algorithms. W3, LF/HF and Mean RR were selected by three algorithms. Acc Capacity, Guzik, DFA a1, AttEn, FuzzyEn, W0 and LF abs were not selected by any algorithm.

The set of features selected for each machine learning algorithm was used to train the respective classification model, where the Rassi score risk class was the output. Table 4 shows the performance of the algorithms for the best set of parameters found. The best general performance was achieved by SVM, while the worst was obtained by RF.

**Table 4:** Performance of the classification models created to predict the Rassi risk group of each patient

	Precision				Recall				F1-score				Acc
	Low	Inter	High	Avg	Low	Inter	High	Avg	Low	Inter	High	Avg	
KNN	0.62	0.29	0.43	0.44	0.81	0.30	0.19	0.43	0.70	0.29	0.26	0.42	0.48
SVM	0.73	0.55	0.58	0.62	0.85	0.55	0.44	0.61	0.79	0.55	0.50	0.61	0.65
MLP	0.66	0.58	0.43	0.55	0.73	0.55	0.38	0.55	0.69	0.56	0.40	0.55	0.58
RF	0.59	0.29	0.25	0.38	0.65	0.25	0.25	0.38	0.62	0.27	0.25	0.38	0.42

KNN: *k*-nearest neighbors; SVM: support vector machine; MLP: multilayer perceptron; RF: random forest; Low: low-risk group; Inter: intermediate-risk group; High: high-risk group; Acc.: accuracy; avg: average over the three groups

**Fig. 2:**
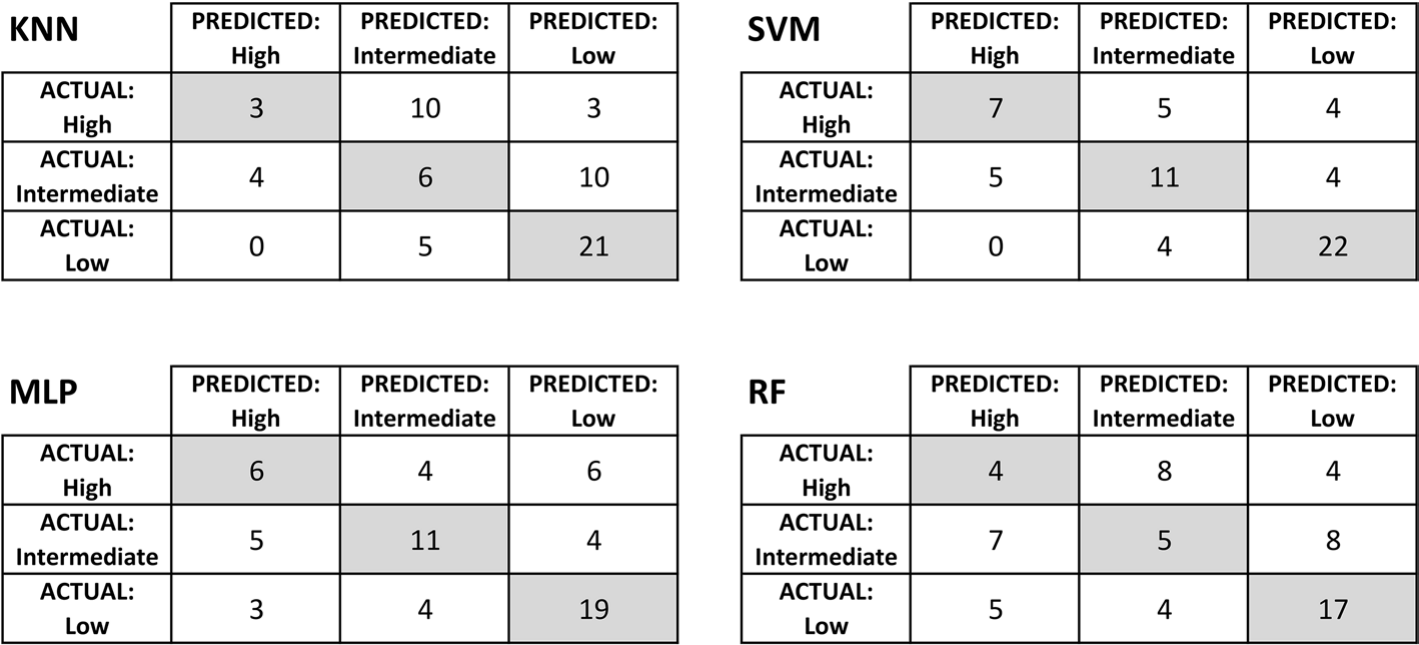
Confusion matrices for the best classification models (same shown in Table 4). KNN: *k*-nearest neighbors; SVM: support vector machine; MLP: multilayer perceptron; RF: random forest. Low: low-risk group; Intermediate: intermediate-risk group; High: high-risk group

Figure 2 shows the confusion matrices of the models reported in Table 4. The figure shows that the class distribution is unequal, with 16, 20, and 26 examples in the “High”, “Intermediate”, and “Low” classes, respectively. Despite the data set being a little unbalanced, no model resulted in a biased solution of voting only in the majority class (“Low”). The worse performance was obtained by KNN and RF, which missed most of the patients in the “High” and “Intermediate” classes, incorrectly assigning them to the “Intermediate” and “Low” classes, respectively. Classification performance of patients in the “Low” risk group was good in all classifiers (F1-score between 0.62 and 0.79, as shown in Table 4).

## Discussion

### HRV and the risk of death in Chronic Chagas Cardiomyopathy (CCC)

In this study we evaluated the association of HRV indices, singly or combined, with a risk marker for overall mortality in individuals with CCC. Taking the HRV indices singly, we found that patients with CD at higher risk of death, as predicted by the Rassi score, present a more fragmented heart rate and a lower phase entropy. In other words, CCC patients with increased heart rate fragmentation and decreased phase entropy have a higher probability of death. Heart rate fragmentation is a recent approach that captures ultra-fast oscillatory profiles of heart rate, in a range of frequency which is not under the exclusive cardiac parasympathetic control. Increased heart rate fragmentation (e.g., PIP and W3) has been shown in individuals with coronary artery disease [16], as well as in other cardiovascular disturbances [17], but its mechanism is still under investigation. In those conditions it is postulated that both intrinsic molecular alterations in the sinus node pacemaker cells and derangements of their autonomic control may play a role [18, 19]. The infection with *T. cruzi* causes damage to all structures of the heart through cell toxicity and inflammation, including the cardiac intrinsic autonomic innervation, the exciting-conducting system and the contractile fibers [11]. Hence, the finding of an increased heart rate fragmentation in patients with CCC is probably a direct consequence of both dysautonomia and cardiac tissue damage that entails a high risk of death as signaled by the Rassi score. On the other hand, entropy methods, in general, rely on the calculation of the information or information rate generated by time series. The differences among approaches are mostly related to how the probabilities of events of interest are estimated. In phase entropy the definition of such events and the estimation of their probabilities are considerably more intricate compared to other approaches, making its practical interpretation even more complex [20]. Therefore, since in our study a decreased phase entropy was not accompanied by alterations in the other entropy approaches, it becomes impossible for us to provide a reasonable interpretation of such a finding.

Previous studies have reported HRV alterations in patients with CD, usually comparing a population of healthy subjects with patients in the chronic phase of CD. Some studies focused on distinct findings in individuals with the indeterminate, cardiac and digestive forms of CD [21–27], while others grouped the patients according to their LV systolic function to stratify the different levels of cardiac involvement [28, 29]. Interestingly, findings from all studies agree that classic linear time- and frequency-domain HRV indices, such as SDNN, RMSSD and the absolute powers at LF and HF bands, are decreased in CD. Those studies corroborated the results of earlier investigations that showed impaired chronotropic cardiovascular responsiveness to autonomic tests, such as the head-up tilt, handgrip, Valsalva maneuver and intravenous infusion of vasoactive drugs, which were shown to be decreased in individuals with various forms of CD [21, 30, 31]. In addition, to the best of our knowledge, only one study evaluated HRV changes among the three Rassi score risk groups. Merejo-Peña et al. [32] described that absolute powers at LF and HF bands are similar among risk groups when individuals are assessed at rest (comparable to our study design), while LF/HF ratio decreased with increasing Rassi score risk. Our results agree with their initial findings. However, with our more thorough approach of HRV, we extended and amplified the scope when we showed that LF and HF absolute powers did not change among risk groups, but there was a tendency of lower LF/HF ratio when the Rassi score risk increases.

When HRV indices were combined, using machine learning algorithms to predict the patients’ risk group, richer results were found in comparison to the analyzes using HRV indices taken singly. The best risk prediction model (SVM) achieved an averaged F1-score of 0.61 and an accuracy of 0.65. This performance, as can be demonstrated in the confusion matrix, is not due to a biased learning, such as memorizing the majority class. Of note, the worst performance was obtained by KNN and RF, which wrongly classified most of the individuals in the intermediate- and high-risk groups. In fact, from their confusion matrices, one can notice that most individuals from the actual high-risk group were classified as intermediate-risk, while most individuals from the actual intermediate-risk group were classified as low-risk. Those results show a tendency for these classifiers to underestimate the real risk of patients in general. Although it could be a consequence of the slightly higher number of samples belonging to the low-risk group, we hypothesize this is indeed due to the complexity of the problem, where a big variety of patterns may be present in the same risk-class. Thus, we believe that increasing the sample size has the potential to improve the classification performances.

The feature selection step, performed prior to the creation of the final predictive models, showed that no HRV index was selected by all the four machine learning schemes. However, excluding KNN and RF, which showed poor classification performances, we found that LF/HF ratio and mean RR were features selected by both SVM and MLP, while the indices that were abnormal among the risk groups (PhaseEn and PIP) were not selected by any of these two algorithms. Only W3, which showed borderline significance among groups, was selected by MLP. Those results demonstrate the importance of combining markers for the prediction of complex variables. Unfortunately, virtually all studies that assessed HRV in individuals with CD evaluated HRV indices taken singly. An important exception was the study by Alberto and co-workers [33], which evaluated the combination of eight HRV indices (time-domain, acceleration/deceleration capacity and heart rate turbulence) to create predictive models of sudden death in patients with CD. Their best models showed accuracy near 90%, demonstrating the high potential of combining different HRV methods for the risk assessment in CD.

One of the leading causes of death in CD is sudden cardiac death, where the most common underlying mechanism is the occurrence of sustained ventricular tachycardia or fibrillation [6, 8, 9, 32, 34, 35]. Although ventricular fibrillation is causally associated with widespread cardiac fibrosis induced by the persistent infection by *T. cruzi*, cardiac dysautonomia is also likely to play an important role to trigger sudden death in patients with CCC. Although the intrinsic mechanism, whereby autonomic impairment increases susceptibility to life threatening arrhythmias is still unclear in CD, it is plausible to consider that it may lead to abnormal excitability and conductibility properties of cardiomyocytes [9, 11, 32, 36–39]. Moreover, although impairment of parasympathetic control of the sinus node is well described in CD [40, 41], the effects of cardiac denervation associated with the *T. cruzi* infection are more complex at the ventricular level. This aspect is not directly evaluated in our study despite the plethora of indices assessing HRV, because they mostly reflect the autonomic control of the sinus node. Hence, although catecholamine-induced cardiomyopathy has not been technically proven to exist in the context of Chagas disease, a sympatho-vagal imbalance of the heart may configure an important factor for triggering sudden cardiac death [39]. In addition, studies of our group using MIBG-based techniques to assess the myocardial sympathetic system have shown striking abnormalities of catecholamine uptake that correlate with the intensity of malignant ventricular arrhythmia in patients with CCC [42–44]. In addition, Souza et al. suggested that QT interval dispersion is a good risk predictor of sudden death in patients with CCC, differently from the Rassi score which refers to overall mortality [35]. The QT interval dispersion is defined by the difference between the maximum and minimum QT intervals calculated within leads in a conventional 12‐lead ECG [45]. This concept is different from QT interval variability, where beat-by-beat series of QT intervals are generated and some properties are estimated over this series, such as the standard deviation and LF and HF components [46]. Augmented QT variability has been suggested to be linked to sympathetic activation in normal subjects [46] and its role in the prognostication of CD warrants further investigation in the future.

### Digestive involvement and HRV

Depopulation of the intramural autonomic innervation causing dysfunctional motility is considered the main mechanism leading to megaesophagus and megacolon of CD etiology [47]. Although the essential pathogenetic mechanism causing the appearance of CCC involves myocardium necrosis and its replacement with fibrotic components, impairment of the autonomic control of the heart is also a hallmark of cardiac involvement in CD [2]. In the past a few investigations reported on abnormal autonomic control of the sinus node in patients with the isolated digestive form of CD, i.e., without clinical signs of cardiac involvement [30, 48]. While this outcome highlights the fact that severe dysautonomia at the sinus node level does not per se lead to the full-blown syndrome of CCC, the possibility that patients with the mixed form of CD could have a more severe degree of autonomic impairment than those with isolated CCC (without megaesophagus or megacolon) had not been explored previously. In our study this hypothesis was directly tested, being confirmed by the comparison of HRV indices between patients with the isolated cardiomyopathy and mixed forms of CD, i.e., there was a decreased TINN and a strong tendency for decreased power of HRV spectra at LF band in patients with the cardiodigestive form. Decreased absolute power at the low-frequency band (LF abs) without alterations in HF power (also in absolute units) and RMSSD is strongly suggestive of decreased cardiac sympathetic modulation. On the other hand, TINN is a geometrical approximation of the cardiac interval histogram base, representing the overall HRV, although more influenced by the lower than higher frequencies [49]. Altogether, those results suggest that there is decreased sympathetic modulation of the sinus node in patients with the mixed form of CD as compared to CCC.

It is noteworthy that cardiac parasympathetic denervation is the most frequently reported autonomic derangement in patients with CD, being directly ascribed to the neuronal depopulation of the cardiac intramural ganglia and by other mechanisms interfering with visceral afferents from the thorax and abdomen. The autonomic impairment can also elicit changes in HRV, either directly modulating the cardiac vagal activity or indirectly affecting neural networks related to the HR regulation, such as the baroreflex central pathway [50]. This is corroborated by our findings, since although HRV indices classically attributable to the influence of parasympathetic control were not altered (HF abs and RMSSD), we cannot ignore the influence of the parasympathetic nervous system on LF abs. It is recognized that both sympathetic and parasympathetic neural discharges have oscillatory components at low and high frequency bands [51]. However, due to the considerably slow beta-adrenergic signaling, which fully depends on the formation of second messengers, compared to the muscarinic (cholinergic) signaling, the high-frequency sympathetic modulation is not effectively transduced to heart rate [52]. This is the reason why the high-frequency oscillations of heart rate are considered exclusively an effect of parasympathetic modulation (coupled to the respiration), whereas the low-frequency variations of heart rate can be the result of both sympathetic and parasympathetic influences.

Sympathetic denervation was also clearly demonstrated in anatomical and functional studies of animal models of *T. cruzi* infection [53] and in humans with CD [2, 40, 41]. While these studies indicate the impairment of sinus node adrenergic regulation, it is even more relevant to mention that the abnormal sympathetic regulation in individuals with CCC also exists at the ventricular level, and possibly play an important role in triggering ventricular arrhythmias in those individuals [44]. Thus, the findings of our study for the first time suggest that patients with the cardiodigestive form of CD have a higher degree of both sympathetic and parasympathetic cardiac denervation compared with patients with CCC alone.

## Limitations

Our study has various limitations. First, the high percentage of patients in use of betablockers, amiodarone, diuretics and ACE inhibitors may configure a bias, since those medications have a high potential to affect HRV [54–56]. However, except for amiodarone in the low-risk group, all groups showed a comparable prevalence of using those medications. Moreover, this sample represents the real scenario in a population with CD, where risk scores and prognostic evaluations are often studied. Second, the sample size used is admittedly low, so are the power values of the statistical tests (between 0.05 and 0.52). It means that the groups evaluated here might have more real differences than the ones presented. However, the enrollment of patients with all the necessary criteria is a challenging task. From the initial 134 patients, we ended up with only 31. To diminish the effect of this low sample size for the machine learning algorithms, we split the HRV series into two segments. Since the series size allowed that and this procedure represents an increase in the sample size with real data, it is preferable to methods of synthetic data augmentation. Third, the timeframe from the ECG recordings to the other exams (echocardiography and Holter) may introduce some bias to the Rassi score. The ideal scenario is the one where all patients have their exams obtained on the same day or in a very short timeframe from the ECG, avoiding influences of the disease evolution on the findings. Nevertheless, the median timeframe was 5.8 (echocardiogram) and 5.1 (Holter) months, which we believe is quite acceptable considering the slow evolution of the symptoms of the general population with CCC. Fourth, this study evaluated the HRV during baseline rest conditions only. However, it is recognized that the responsiveness of the HRV to cardiovascular challenges, such as postural maneuvers, can provide more information about the autonomic control, usually not observed during rest conditions [57]. Finally, the set of HRV indices explored here, although comprehensive, is not extensive. The prognostic value of several other entropy [58–60], fractal [61] and general [62–64] methods in CCC should be investigated in future studies.

## Conclusions

Our findings show that increased heart rate fragmentation indices are associated with worsening prognosis as assessed with the Rassi score risk for overall mortality in patients with the cardiomyopathy form of CD. Moreover, the combination of HRV indices to predict the Rassi score risk class of individuals with CCC achieved good performances (F1-score of 0.61 and accuracy of 0.65). Due to the apparent complexity of this classification problem, it is plausible to assume that this performance can be further improved with a higher sample size, where the variety of patterns could be more adequately expanded for each risk group. Finally, individuals with the cardiodigestive form of CD showed a decrease in the slow oscillatory components of heart rate when compared with individuals with isolated CCC, a finding possibly explained by a higher degree of sympathetic and parasympathetic cardiac denervation in individuals with the mixed form of CD.

## Methods

### Population

The patients included in the study had to fulfill three basic criteria: (a) age >  = 18 years; (b) to have an established diagnosis of CCC; c) to sign an informed consent. A total of one hundred and thirty-four (134) patients with CD were initially recruited between May 2019 and March 2020 from the University Hospital of Ribeirão Preto Medical School, University of São Paulo, as described in a previous study [15]. All patients agreed spontaneously to participate in the study and signed a written term of informed consent. The study was approved by the Research Ethics Committee of the University Hospital under Protocol #3308377.

From the total enrolled patients, 50 were excluded due to the presence of non-sinus rhythm or an unacceptable number of arrhythmic events (> 5% of the total number of beats in the study; see section 6.5). Another 40 patients were excluded due to the lack of all necessary exams to assess the Rassi score in a timeframe of 17 months around the date of inclusion in the study (see section 6.3); finally, 13 additional patients were ineligible for lacking the criterium of a diagnosis of CCC (see section 6.2). Patients with an implanted cardioverter–defibrillator were allowed when confirmed in sinus rhythm during the ECG recordings (2 cases). Therefore, thirty-one (31) patients were eligible for the study. Table 1 shows the demographic and clinical variables of the included patients.

### Definition of chronic cardiomyopathy form of Chagas disease

According to the Second Brazilian Consensus on Chagas Disease [5], the CCC form of CD can be defined by the presence of ECG alterations suggestive of typical CD cardiac involvement. According to the consensus and other supportive studies, typical ECG alterations in CD are [5, 65, 66]: bradycardia (heart rate < 40 bpm); low QRS voltage; intraventricular conduction disorders (right bundle branch block, left anterior–superior fascicular block, posterior–inferior fascicular block, left bundle-branch block); atrioventricular block (first degree, second degree or complete); diffuse alteration of ventricular repolarization; QT interval prolongation (QTc > 440 ms for men or QTc > 460 ms for women); ventricular extrasystoles (isolated, polymorphic or paired); ventricular bigeminism or trigeminism; sustained or nonsustained ventricular tachycardia; variable or lack of P wave (wandering atrial pacemaker, atrial flutter, atrial tachycardia, atrial fibrillation or junctional rhythm).

Thus, the 31 patients of the study had at least one of these ECG abnormalities being considered to have the CCC form of CD. The 31 patients with CCC included were also evaluated for the presence of typical symptoms and/or objective signs of digestive organic involvement usually detected in patients with CD. These typical symptoms included dysphagia, esophageal pain, intestinal constipation and abnormalities detected in the esophagus or colon during radiological, endoscopy, colonoscopy, or motility studies.

### Rassi score calculation

The Rassi score was developed and validated to quantify the overall risk of death for patients with the CCC form of CD [34]. It is based on ascribing points to 6 demographical, clinical, echocardiographic, electrocardiographic and radiologic characteristics bearing significant prognostic information, namely, New York Heart Association (NYHA) functional class III or IV (5 points), cardiomegaly on chest radiography (5 points), segmental or global wall-motion abnormality on echocardiography (3 points), nonsustained ventricular tachycardia on 24-h Holter monitoring (3 points), low QRS voltage on conventional 12-lead ECG (2 points), and male gender (2 points). The highest risk occurs when the Rassi score is 20 (all risk factors are present), while the lowest risk occurs when the Rassi score is 0 (no risk factor present) [34].

Although cardiomegaly is classically defined by a cardiothoracic ratio higher than 0.5 in the plain chest X-ray image, it has been recently shown that left ventricular end-diastolic diameter (LVEDD) higher than 60 mm can be considered a good surrogate for cardiomegaly in patients with CD [67]. Thus, in our study, the presence of cardiomegaly in the assessment of the Rassi score was defined when LVEDD was > 60 mm. The echocardiogram was obtained within 5.8 [3.2, 7.7] months from the ECG recordings, while the 24 h Holter monitoring was recorded within 5.1 [3.3, 8.5] months (median [1st, 3rd] quartiles).

The 31 patients in the study were grouped according to their prognoses as defined by the following ranges of Rassi score [34]: low-risk group (0 to 6 points), intermediate-risk group (7 to 11 points), and high-risk group (12 to 20 points). Table 1 shows the demographic and clinical variables of eligible patients grouped by the Rassi score risk classes.

### Electrocardiographic recordings

The analysis of the ECG recordings was performed as previously described [15]. In summary, all patients were subjected to two ECG recordings: the conventional 12-lead ECG and a single-lead (DI, DII or DIII) ECG. The two recordings were obtained on the same day, in a dedicated recording room at the Cardiology Division of the University Hospital, Ribeirão Preto Medical School, University of São Paulo.

The conventional 12-lead ECG was recorded using the conventional routine equipment from the hospital clinical facilities allowing the identification of abnormalities typical of the CCC (see section 6.2). The single-lead ECG was recorded during 10–20 min using a portable device (PowerLab, ADInstruments, Australia) at 1 kHz for subsequent HRV analysis. Patients were allowed 2 min of rest (stabilization period) before the recording started and were instructed not to talk and to breathe calmly and spontaneously during the whole recording.

### Data processing

The single-lead ECG recordings were processed using computer software (LabChart Pro, ADInstruments, Australia) to detect the ECG R-peaks and create RR series, which correspond to the time intervals between successive R-peaks. Artifacts were removed from RR series using a moving median procedure implemented in PyBioS software [68]. Briefly, the RR series’ baseline was estimated using a moving median of size *win*. Next, an upper and lower threshold was defined as the baseline shifted up and down by a tolerance factor (*tol*). This tolerance factor represents the percentage of the baseline’s mean value. Finally, RR intervals lying below the lower or above the upper thresholds are removed (no replacement) from the series. The optimal values of *win* and *tol* were manually chosen for each RR series, varying in the range win = [6, 30] and tol = [0.02, 0.15]. When the correction required more than 5% of removals, i.e., the number of beats to be removed was higher than 5% of the estimated number of beats, the patient was excluded from the study (exclusion criterion as outlined above). The median [1^st^, 3^rd^] quartiles of removals percentage was 0.8 [0.2, 2.3] and the size of the corrected RR series was 971 [827, 1121] samples.

Since machine learning analysis requires a reasonable number of samples to create good prediction models, all RR series were split into two equally sized segments, corresponding to a period of 5–10 min of the ECG recording. Then, HRV indices were estimated separately from the two segments (*N* = 62). This procedure is preferable to creating spurious data with augmentation techniques. In addition, to avoid the bias of using different data sets, the same duplicated data was used for comparisons among the three Rassi score risk groups. Both analyses (machine learning and group risk comparisons) considered the duplication of data from each subject, as described in sections 6.7 and 6.8.

### Heart rate variability

Thirty HRV indices were calculated from the RR series. They were derived from different families of methods, inspired by classical linear approaches, such as the statistical and spectral indices, as well as methods derived from nonlinear dynamics.

The acceleration (Acc) and the deceleration (Dec) capacity of heart rate were calculated as described by Bauer et al. [69]. Essentially, Acc and Dec capacity estimate the average magnitude of increases and decreases in heart rate. However, as the HRV series are represented by RR interval series, accelerations and decelerations refer to decreases and increases of RR intervals, respectively, both in milliseconds. From the family of asymmetry indices, three approaches were selected, namely, Porta’s, Guzik’s and Ehlers’ indices [70]. Asymmetry indices evaluate whether the positive changes in RR intervals are similar to the negative changes. Porta’s and Guzik’s indices are given as a percentage value, where 50% characterizes series which are time-reversible. In contrast, Ehlers’s index is dimensionless and values equal to zero characterize time-reversible series. The fractal scaling of RR series was estimated by detrended fluctuation analysis (DFA) [71]. Since RR series are short, only the short-term scaling exponent (a1) was calculated, in the range of window sizes from 5 to 15 [15].

From the wide family of entropy methods, seven were utilized, namely, attention entropy (AttEn) [72], dispersion entropy (DispEn), with *m* = 3 and *c* = 6 [73], distribution entropy (DistEn), with *m* = 3 and *M* = 512 [74], fuzzy entropy (FuzzyEn), with *m* = 2, *r* = 0.15 and *n* = 2 [75], permutation entropy (PermEn), with *m* = 3 [76], phase entropy (PhaseEn), with *k* = 16 [20] and sample entropy (SampEn), with *m* = 2 and *r* = 0.15 [77]. Entropy methods, in general, quantify the irregularity or unpredictability of RR intervals so that the higher the entropy, the more irregular or unpredictable is the RR series. The heart rate fragmentation was estimated by the total percentage of inflection points (PIP) and by the percentage of patterns with zero (W0), one (W1), two (W2) and three (W3) inflection points [16, 78]. Each pattern represents a sequence of four consecutive RR interval differences so that one pattern can contain, at most, three inflection points. The symbolic dynamics method proposed by Porta et al. [79] was also calculated. In this case, RR intervals are quantized into six levels (symbolization) and patterns are created as sequences of three consecutive symbols; then, all patterns are classified into one of the following families: 0 V (zero variations), 1 V (one variation), 2LV (two-like variations) or 2UV (two-unlike variations). Previous studies reported that the percentage of 0 V patterns is related to the cardiac sympathetic modulation, whereas the percentage of 2UV patterns is linked to the cardiac parasympathetic modulation [79, 80].

Classical linear time- and frequency-domain HRV indices were also estimated. Frequency-domain components were estimated from the power spectral density of RR series, calculated using the modified periodogram [81]. In short, RR series were interpolated (by cubic spline) and resampled at 3 Hz. Next, series were segmented into windows of 512 samples, with 50% overlap, and a Hanning windowing was applied to each segment to attenuate the spectral leakage. The power spectrum was estimated from each segment and the powers at low- (LF: 0.04 to 0.15 Hz) and high-frequency (HF: 0.15 to 0.40 Hz) bands were integrated. The median absolute (abs) power at LF and HF bands (in ms^2^), as well as the LF/HF ratio (dimensionless), over all segments, were eventually used. The absolute power at HF band is considered an important marker of cardiac vagal modulation, driven by respiration. In contrast, the absolute power at LF band represents both sympathetic and parasympathetic cardiac modulation [49, 82]. From time-domain, five HRV indices were calculated, namely, the mean RR interval, standard deviation of RR intervals (SDNN), root mean square of the successive differences in the RR series (RMSSD), triangular index (Triang Index) and triangular interpolation of RR interval histogram (TINN) [49].

### Classical statistical analysis

The Gaussian distribution of HRV indices was checked using the Shapiro–Wilk test. Since most indices did not pass the normality test, data were transformed prior to the statistical comparisons using the Yeo-Johnson power transformation [83]. The *lambda* of the transformation that maximizes the log-likelihood function was chosen for each HRV index. Original raw values (not transformed) are reported as median [1st, 3rd] quartiles.

Differences of HRV indices among the risk groups (“Low”, “Intermediate” and “High”) or between the disease forms (“Cardiomyopathy” and “Cardiodigestive”) were evaluated using a mixed-effects ANOVA, where the two HRV measurements from each subject represent the within-subjects factor and the risk (or disease form) groups represent the between-subjects factor. It must be emphasized that we were not focused on the possible differences within-subjects, nor in its interaction with each group. Only the between-subjects factor, i.e., the risk class or disease form, was evaluated. When statistical significance was found in the ANOVA, pairwise comparisons were performed using Tukey's post-hoc test. Significance was considered when *p* < 0.05.

For the sake of comparison, we performed the same tests using the whole RR series to estimate the HRV indices (data not shown). In this case, only one HRV index was estimated for each subject and the one-way ANOVA was applied to compare the three groups. Results (*p* values) are fairly the same as the presented here.

### Machine learning modeling

Classification models were created to predict the Rassi risk class (output) of each patient, with their HRV indices taken as inputs. Four machine learning classifiers were used, representing some of the main paradigms used in machine learning: *k*-nearest neighbors (KNN), support vector machine (SVM), multilayer perceptron (MLP) and random forest (RF). All analyzes were performed using the *scikit-learn* library for Python [84]. HRV indices were normalized to mean = 0.0 and SD = 1.0 prior to the analysis.

#### Cross-validation scheme

A *k*-fold cross-validation scheme was adopted in feature selection (fivefold) and training (tenfold) steps. Folds were carefully created to ensure that: (1) the two HRV indices obtained from the same subject always be in a single fold, so that data from the same subject is never used for both train and test; and (2) approximately the same proportion of classes in all folds (stratified folds).

#### Feature selection

Since there are too many HRV indices (features) in comparison to the sample size, and that some of the HRV indices may not be relevant in the classification problems, a search for the best set of features was performed for each machine learning algorithm using a Sequential Feature Selection scheme. Essentially, an iterative process that greedily adds (forward selection) or removes (backward selection) features to create a good subset of features is generated. At each step, the best feature to add or remove is found based on the cross-validation (fivefold) score obtained by the investigated classifier. The process is repeated until the number of desired features for the classifier is selected. The parameters of each classifier during feature selection were initially set as default. When the best set of parameters found by Grid Search (see next section) was different from the default of the *scikit learn* library, feature selection was performed again using the new set of parameters to check whether the newly selected set of features provide a better classification.

Forward search starts with an empty set of features and, at each step, greedily adds the feature that provides the highest increase in the performance of the classifier. In contrast, backward search starts with all HRV features and iteratively removes the feature that provides the highest increase in the performance of the classifier. In this study, both forward and backward selection were applied, setting the desired number of features to 15 (half of the total). Only features selected at both forward and backward searches were taken as the final selected features.

#### Training

The subset of optimal features found for each classifier was used to train the respective classifier for the prediction of the Rassi score risk group of each patient. The best set of parameters for each classifier was found using a Grid Search Cross-Validation (tenfold) scheme, varying the following parameters: number of neighbors (1 to 20) and weights (uniform, distance) for KNN; kernel (linear, rbf), C and gamma (0.001, 0.01, 0.1, 1, 10, 100) for SVM; hidden layers (15, 30, 50, 70, 100, 150), activation function (tanh, relu), solver (lbgfs, adam), maximum iterations (200, 500, 1000, 2000, 5000) and alpha (0.001, 0.01, 0.1, 1.0) for MLP; number of estimators (15, 30, 50, 70, 100, 150, 200, 250, 300) for RF. Details about those parameters are found in the *scikit-learn* documentation [85].

The ability of each classification model to predict the correct class of risk (“Low”, “Intermediate” or “High”) was evaluated by precision, recall, F1-score and accuracy. While precision is defined as $$TP/(TP+FP)$$, recall is defined as $$\text{TP}/(\text{TP}+\text{FN})$$, where $$\text{TP}$$, $$\text{FP}$$ and $$\text{FN}$$ represent the true positive, false positive and false negative of classifications, respectively. The F1-score combines these two previous scores in a single metric, defined as $$[2*\text{precision}*\text{recall}/(\text{precision}+\text{recall)}]$$. Those scores are shown for each class (one against all) and as the average over the three classes. The accuracy quantifies the ratio between the number of corrected classified samples (no matter the class) to the total number of samples. The confusion matrices of each classifier are also shown.

## Data Availability

The data sets gathered and/or analyzed during the current study are available from the corresponding author on reasonable request.

## References

[1] Chagas C (1909). Nova tripanozomiaze humana: estudos sobre a morfolojia e o ciclo evolutivo do *Schizotrypanum**cruzi* n. gen., n. sp., ajente etiolojico de nova entidade morbida do homem. Mem Inst Oswaldo Cruz.

[2] Marin-Neto JA, Cunha-Neto E, Maciel BC, Simões MV (2007). Pathogenesis of chronic Chagas heart disease. Circulation.

[3] Rassi A, Marin Neto JA, Rassi A, Rassi A, Marin Neto JA, Rassi A (2017). Chronic Chagas cardiomyopathy: a review of the main pathogenic mechanisms and the efficacy of aetiological treatment following the BENznidazole Evaluation for Interrupting Trypanosomiasis (BENEFIT) trial. Mem Inst Oswaldo Cruz.

[4] Dutra WO, Menezes CAS, Magalhães LMD, Gollob KJ (2014). Immunoregulatory networks in human Chagas disease. Parasite Immunol.

[5] Dias JCP, Ramos AN, Gontijo ED, Luquetti A, Shikanai-Yasuda MA, Coura JR (2016). 2nd Brazilian Consensus on Chagas Disease, 2015. Rev Soc Bras Med Trop.

[6] Rassi A, Rassi A, Rassi SG (2007). Predictors of mortality in chronic Chagas disease: a systematic review of observational studies. Circulation.

[7] Simões MV, Romano MMD, Schmidt A, Martins KSM, Marin-Neto JA (2018). Chagas disease cardiomyopathy. Int J Cardiovasc Sci.

[8] Rassi A, Rassi SG, Rassi A (2001). Sudden death in Chagas’ disease. Arq Bras Cardiol.

[9] Keegan R, Yeung C, Baranchuk A (2020). Sudden cardiac death risk stratification and prevention in chagas disease: a non-systematic review of the literature. Arrhythm Electrophysiol Rev.

[10] Amorim DD, Marin Neto JA (1995). Functional alterations of the autonomic nervous system in Chagas’ heart disease. Sao Paulo Med J.

[11] Junqueira LF (2012). Insights into the clinical and functional significance of cardiac autonomic dysfunction in Chagas disease. Rev Soc Bras Med Trop.

[12] Acharya UR, Joseph KP, Kannathal N, Lim CM, Suri JS (2006). Heart rate variability: a review. Med Biol Eng Compu.

[13] Huikuri HV, Stein PK (2013). Heart rate variability in risk stratification of cardiac patients. Prog Cardiovasc Dis.

[14] Voss A, Schulz S, Schroeder R, Baumert M, Caminal P (2009). Methods derived from nonlinear dynamics for analysing heart rate variability. Phil Trans R Soc A.

[15] Silva LEV, Moreira HT, Bernardo MMM, Schmidt A, Romano MMD, Salgado HC (2021). Prediction of echocardiographic parameters in Chagas disease using heart rate variability and machine learning. Biomed Signal Process Control.

[16] Costa MD, Davis RB, Goldberger AL (2017). Heart rate fragmentation: a new approach to the analysis of cardiac interbeat interval dynamics. Front Physiol.

[17] Costa MD, Redline S, Davis RB, Heckbert SR, Soliman EZ, Goldberger AL (2018). Heart rate fragmentation as a novel biomarker of adverse cardiovascular events: the multi-ethnic study of atherosclerosis. Front Physiol.

[18] Lensen IS, Monfredi OJ, Andris RT, Lake DE, Moorman JR (2020). Heart rate fragmentation gives novel insights into non-autonomic mechanisms governing beat-to-beat control of the heart’s rhythm. JRSM Cardiovasc Dis.

[19] da Silva TM, Silva CAA, Salgado HC, Fazan R, Silva LEV (2021). The role of the autonomic nervous system in the patterns of heart rate fragmentation. Biomed Signal Process Control.

[20] Rohila A, Sharma A (2019). Phase entropy: a new complexity measure for heart rate variability. Physiol Meas.

[21] Guzzetti S, Iosa D, Pecis M, Bonura L, Prosdocimi M, Malliani A (1991). Impaired heart rate variability in patients with chronic Chagas’ disease. Am Heart J.

[22] Emdin M, Marin-Neto JA, Carpeggiani C, Maciel BC, Macerata A, Pintya AO (1992). Heart rate variability and cardiac denervation in Chagas’ disease. Heart rate variability and cardiac denervation in Chagas disease 5th ed. J Ambulat Monitor.

[23] Rassi A, Rassi SG, Waktare JEP, Rassi AG, Malik M, Rassi A (1999). Cardiopatia chagásica crônica: significado prognóstico da variabilidade da freqüencia cardíaca no domínio do tempo. Arq Bras Cardiol.

[24] Ribeiro ALP, Moraes RS, Ribeiro JP, Ferlin EL, Torres RM, Oliveira E (2001). Parasympathetic dysautonomia precedes left ventricular systolic dysfunction in Chagas disease. Am Heart J.

[25] Vasconcelos DF, Junqueira LF (2009). Distinctive impaired cardiac autonomic modulation of heart rate variability in chronic Chagas’ indeterminate and heart diseases. J Electrocardiol.

[26] Barbosa-Ferreira JM, Mady C, Ianni BM, Lopes HF, Ramires FJA, Salemi VMC (2015). Dysregulation of autonomic nervous system in Chagas’ heart disease is associated with altered adipocytokines levels. PLoS ONE.

[27] Vizcardo M, Ravelo A. Use of Approximation Entropy for Stratification of Risk in Patients With Chagas Disease. 2018 Computing in Cardiology Conference (CinC). 2018. p. 1–4.

[28] Ribeiro ALP, Lombardi F, Sousa MR, Lins Barros MV, Porta A, Costa Val Barros V (2002). Power-law behavior of heart rate variability in Chagas’ disease. Am J Cardiol.

[29] Ribeiro ALP, Schmidt G, Sousa MR, Lombardi F, Gomes MED, Perez AA (2003). Heart rate turbulence in Chagas disease. Pacing Clin Electrophysiol.

[30] Sousa AC, Marin-Neto JA, Maciel BC, Gallo Júnior L, Barreto-Martins LE, Amorim DS (1987). Use of isometric exercise to demonstrate cardiac parasympathetic impairment in the digestive form of Chagas’ disease. Braz J Med Biol Res.

[31] Marin-Neto JA, Bromberg-Marin G, Pazin-Filho A, Simões MV, Maciel BC (1998). Cardiac autonomic impairment and early myocardial damage involving the right ventricle are independent phenomena in Chagas’ disease. Int J Cardiol.

[32] Merejo Peña CM, Reis MS, de Pereira BB, do Nascimento EM, Pedrosa EC (2018). Dysautonomy in different death risk groups (Rassi score) in patients with Chagas heart disease. Pacing Clin Electrophysiol.

[33] Alberto AC, Pedrosa RC, Zarzoso V, Nadal J (2020). Association between circadian Holter ECG changes and sudden cardiac death in patients with Chagas heart disease. Physiol Meas.

[34] Rassi A, Rassi A, Little WC, Xavier SS, Rassi SG, Rassi AG (2006). Development and validation of a risk score for predicting death in Chagas’ heart disease. N Engl J Med.

[35] Souza ACJ, Salles G, Hasslocher-Moreno AM, de Sousa AS, Alvarenga Americano do Brasil PE, Saraiva RM (2015). Development of a risk score to predict sudden death in patients with Chaga’s heart disease. Int J Cardiol.

[36] Díaz JO, Mäkikallio TH, Huikuri HV, Lopera G, Mitrani RD, Castellanos A (2001). Heart rate dynamics before the spontaneous onset of ventricular tachyarrhythmias in Chagas’ heart disease. Am J Cardiol.

[37] Benchimol-Barbosa PR, Tura BR, Barbosa EC, Kantharia BK (2013). Utility of a novel risk score for prediction of ventricular tachycardia and cardiac death in chronic Chagas disease—the SEARCH-RIO study. Braz J Med Biol Res.

[38] Hammersley DJ, Halliday BP (2020). Sudden cardiac death prediction in non-ischemic dilated cardiomyopathy: a multiparametric and dynamic approach. Curr Cardiol Rep.

[39] Pedrosa RC (2020). Dysautonomic arrhythmogenesis: a working hypothesis in chronic Chagas cardiomyopathy. Int J Cardiovasc Sci..

[40] Marin-Neto JA, Gallo L, Manço JC, Rassi A, Amorim DS (1975). Postural reflexes in chronic Chagas’s heart disease. Heart rate and arterial pressure responses. Cardiology.

[41] Marin-Neto JA, Gallo L, Manco JC, Rassi A, Amorim DS (1980). Mechanisms of tachycardia on standing: studies in normal individuals and in chronic Chagas’ heart patients. Cardiovasc Res.

[42] Simões MV, Pintya AO, Bromberg-Marin G, Sarabanda AV, Antloga CM, Pazin-Filho A (2000). Relation of regional sympathetic denervation and myocardial perfusion disturbance to wall motion impairment in Chagas’ cardiomyopathy. Am J Cardiol.

[43] Miranda CH, Figueiredo AB, Maciel BC, Marin-Neto JA, Simões MV (2011). Sustained ventricular tachycardia is associated with regional myocardial sympathetic denervation assessed with 123I-metaiodobenzylguanidine in chronic Chagas cardiomyopathy. J Nucl Med.

[44] Gadioli LP, Miranda CH, Pintya AO, de Figueiredo AB, Schmidt A, Maciel BC (2018). The severity of ventricular arrhythmia correlates with the extent of myocardial sympathetic denervation, but not with myocardial fibrosis extent in chronic Chagas cardiomyopathy : Chagas disease, denervation and arrhythmia. J Nucl Cardiol.

[45] Sahu P, Lim PO, Rana BS, Struthers AD (2000). QT dispersion in medicine: electrophysiological Holy Grail or fool’s gold?. Int J Med.

[46] Baumert M, Porta A, Vos MA, Malik M, Couderc J-P, Laguna P (2016). QT interval variability in body surface ECG: measurement, physiological basis, and clinical value: position statement and consensus guidance endorsed by the European Heart Rhythm Association jointly with the ESC Working Group on Cardiac Cellular Electrophysiology. Europace.

[47] Oliveira RB, Ballart C, Abràs A, Gállego M, Marin-Neto JA. Chagas Disease: An Unknown and Neglected Disease. In: Pinazo Delgado M-J, Gascón J, editors. Chagas Disease: A Neglected Tropical Disease [Internet]. Cham: Springer International Publishing; 2020 [cited 2021 Mar 31]. p. 1–26. Available from: 10.1007/978-3-030-44054-1_1

[48] Sousa AC, Marin-Neto JA, Maciel BC, Gallo L, Amorim DS (1987). Cardiac parasympathetic impairment in gastrointestinal Chagas’ disease. Lancet.

[49] Task Force of the European Society of Cardiology and the North American Society of Pacing and Electrophysiology. Heart Rate Variability. Standards of Measurement, Physiological Interpretation, and Clinical Use. Circulation. 1996;93:1043. 8598068

[50] Karemaker JM (2020). Interpretation of heart rate variability: the art of looking through a keyhole. Front Neurosci.

[51] Malliani A, Pagani M, Lombardi F, Cerutti S (1991). Cardiovascular neural regulation explored in the frequency domain. Circulation.

[52] Stauss HM (2003). Heart rate variability. Am J Physiol Regul Integr Comp Physiol.

[53] Alcântara FG (1970). Denervation of the cardiac and cervicothoracic ganglia in Chagas’s disease. Rev Goiana Med.

[54] Kontopoulos AG, Athyros VG, Papageorgiou AA, Skeberis VM, Basayiannis EC, Boudoulas H (1997). Effect of angiotensin-converting enzyme inhibitors on the power spectrum of heart rate variability in post-myocardial infarction patients. Coron Artery Dis.

[55] Tomiyama H, Nakayama T, Watanabe G, Shiojima K, Sakuma Y, Yamamoto A (1999). Effects of short-acting and long-acting loop diuretics on heart rate variability in patients with chronic compensated congestive heart failure. Am Heart J.

[56] Silva HEF, de Almeida RS, Silveira DB, Llaguno M, Resende LAPR, da Silva VJD (2018). Cardiac autonomic modulation and long-term use of amiodarone in patients with chronic Chagasic cardiopathy. Pacing Clin Electrophysiol.

[57] Malik M, Hnatkova K, Huikuri HV, Lombardi F, Schmidt G, Zabel M (2019). CrossTalk proposal: Heart rate variability is a valid measure of cardiac autonomic responsiveness. J Physiol.

[58] Porta A, Baselli G, Guzzetti S, Pagani M, Malliani A, Cerutti S (2000). Prediction of short cardiovascular variability signals based on conditional distribution. IEEE Trans Biomed Eng.

[59] Porta A, De Maria B, Bari V, Marchi A, Faes L (2017). Are nonlinear model-free conditional entropy approaches for the assessment of cardiac control complexity superior to the linear model-based one?. IEEE Trans Biomed Eng.

[60] Borin AMS, Humeau-Heurtier A, Virgílio Silva LE, Murta LO (2021). Multiscale entropy analysis of short signals: the robustness of fuzzy entropy-based variants compared to full-length long signals. Entropy.

[61] Citi L, Valenza G, Purdon PL, Brown EN, Barbieri R. Monitoring heartbeat nonlinear dynamics during general anesthesia by using the instantaneous dominant Lyapunov exponent. Engineering in Medicine and Biology Society (EMBC), 2012 Annual International Conference of the IEEE. IEEE; 2012. p. 3124–7 10.1109/EMBC.2012.6346626 23366587

[62] Maestri R, Pinna GD, Accardo A, Allegrini P, Balocchi R, D’Addio G (2007). Nonlinear indices of heart rate variability in chronic heart failure patients: redundancy and comparative clinical value. J Cardiovasc Electrophysiol.

[63] Silva LEV, Murta LO (2012). Evaluation of physiologic complexity in time series using generalized sample entropy and surrogate data analysis. Chaos.

[64] Bravi A, Longtin A, Seely AJE (2011). Review and classification of variability analysis techniques with clinical applications. Biomed Eng Online.

[65] Castro C, Prata A, Macêdo V (2001). A 13-year clinical study on 190 chronic chagasic patients from Mambaí, Goiás, Brazil. Rev Soc Bras Med Trop.

[66] Garzon SA, Lorga AM, Nicolau JC (1995). Electrocardiography in Chagas’ heart disease. Sao Paulo Med J.

[67] Ramos MRF, Moreira HT, Volpe GJ, Romano M, Maciel BC, Schmidt A (2021). Correlation between cardiomegaly on chest X-ray and left ventricular diameter on echocardiography in patients with chagas disease. Arq Bras Cardiol.

[68] Silva LEV, Fazan R, Marin-Neto JA (2020). PyBioS: a freeware computer software for analysis of cardiovascular signals. Comput Methods Programs Biomed.

[69] Bauer A, Kantelhardt JW, Barthel P, Schneider R, Mäkikallio T, Ulm K (2006). Deceleration capacity of heart rate as a predictor of mortality after myocardial infarction: cohort study. Lancet.

[70] Porta A, Casali KR, Casali AG, Gnecchi-Ruscone T, Tobaldini E, Montano N (2008). Temporal asymmetries of short-term heart period variability are linked to autonomic regulation. Am J Physiol Regul Integr Comp Physiol.

[71] Peng CK, Havlin S, Stanley HE, Goldberger AL (1995). Quantification of scaling exponents and crossover phenomena in nonstationary heartbeat time-series. Chaos.

[72] Yang J, Choudhary GI, Rahardja S, Franti P. Classification of Interbeat Interval Time-series Using Attention Entropy. IEEE Transactions on Affective Computing. 2020;1–1.

[73] Rostaghi M, Azami H (2016). Dispersion entropy: a measure for time-series analysis. IEEE Signal Process Lett.

[74] Li P, Liu C, Li K, Zheng D, Liu C, Hou Y (2015). Assessing the complexity of short-term heartbeat interval series by distribution entropy. Med Biol Eng Comput.

[75] Chen W, Wang Z, Xie H, Yu W (2007). Characterization of surface EMG signal based on fuzzy entropy. IEEE Trans Neural Syst Rehabil Eng.

[76] Bandt C, Pompe B (2002). Permutation entropy: a natural complexity measure for time series. Phys Rev Lett.

[77] Richman JS, Moorman JR (2000). Physiological time-series analysis using approximate entropy and sample entropy. Am J Physiol Heart Circ Physiol.

[78] Costa MD, Davis RB, Goldberger AL (2017). Heart rate fragmentation: a symbolic dynamical approach. Front Physiol.

[79] Porta A, Tobaldini E, Guzzetti S, Furlan R, Montano N, Gnecchi-Ruscone T (2007). Assessment of cardiac autonomic modulation during graded head-up tilt by symbolic analysis of heart rate variability. Am J Physiol Heart Circ Physiol.

[80] Silva LEV, Geraldini VR, de Oliveira BP, Silva CAA, Porta A, Fazan R (2017). Comparison between spectral analysis and symbolic dynamics for heart rate variability analysis in the rat. Sci Rep.

[81] Welch P (1967). The use of fast Fourier transform for the estimation of power spectra: a method based on time averaging over short, modified periodograms. IEEE Trans Audio Electroacoust.

[82] Shaffer F, Ginsberg JP (2017). An overview of heart rate variability metrics and norms. Front Public Health.

[83] Yeo I-K, Johnson RA (2000). A new family of power transformations to improve normality or symmetry. Biometrika.

[84] Pedregosa F, Varoquaux G, Gramfort A, Michel V, Thirion B, Grisel O (2011). Scikit-learn: machine learning in Python. J Mach Learn Res.

[85] API Reference for scikit-learn. 2021. https://scikit-learn.org/stable/modules/classes.html Accessed 31 Mar 2021.

